# Influence of Alloying Elements on the Phase Structure, Stress–Strain Behavior, and Fracture Toughness of Ni_3_Sn: A First-Principles Study

**DOI:** 10.3390/ma18081792

**Published:** 2025-04-14

**Authors:** Haotian Zhang, Jiaoyan Dai, Yinwen Cao, Yanjie Zhang, Mingdong Bao, Yanping Yin

**Affiliations:** 1 Department of Material and Chemical Engineering, Ningbo University of Technology, 201 Fenghua Avenue, Ningbo 315211, China; 2021231115@chd.edu.cn (H.Z.); yingwencao@mail.sdu.edu.cn (Y.C.); yjzhang@nbut.edu.cn (Y.Z.); mdbao@nbut.edu.cn (M.B.); 2 School of Materials Science and Engineering, Chang’an University, No. 75 Changan, Middle Road, Xi’an 710064, China

**Keywords:** intermetallic compounds, alloying elements, toughness, electronic structure, first-principles calculations

## Abstract

Transient liquid-phase bonding (TLPB) enables the low-temperature fabrication of encapsulated solder joints with high-temperature resistance and electromigration resilience; yet, Ni-Sn TLPB joints suffer from brittle fracture due to intermetallic compounds (IMCs). This study investigates the Co, Cu, and Pt alloying effects on Ni_3_Sn via formation energy, molecular dynamics, and first-principles calculations. Occupancy models of Ni_6−x_M_x_Sn_2_ (M = Co, Cu, and Pt) were established, with the lattice parameters, B/G ratios, fracture toughness (K_IC_), and stress–strain behaviors analyzed. The results reveal that Co enhances fracture toughness and reduces Ni_3_Sn anisotropy, mitigating microcrack risks, while Cu/Pt introduce antibonding interactions (Cu–Sn and Pt–Sn), weakening the bonding strength. The classical B/G brittleness criterion proves inapplicable in Ni–M–Sn systems due to mixed bonding (metallic/covalent) and the hexagonal structure’s limited slip systems. The Ni_6−x_Co_x_Sn_2_ formation improves toughness with a low Co content, supported by an electronic structure analysis (density of states and Bader charges). The thermodynamic stability and reduced molar shrinkage (Ni + Sn → Ni_3_Sn) confirm Co’s efficacy in optimizing Ni–Sn solder joints.

## 1. Introduction

Transient liquid-phase bonding (TLPB) packaging technology is characterized by packaging bonding at a lower temperature [1,2,3] and produces solder joints with high melting points for all intermetallic compounds (IMCs), and then solder joints can be used at higher temperatures. IMCs with high melting points have good electromigration resistance [4,5], ensuring the solder joints’ service stability. In the TLPB process, although the formation of intermetallic compounds improves the bonding strength of solder joints, it is easy to cause a brittle fracture due to long-term thermal cycling stress in the subsequent use of solder joints [6,7,8]. Improving the toughness of intermetallic compounds in TLPB is important in order to enhance the brittle fracture resistance of solder joints. In the previous study [9], we compared the mechanical and physical properties of Ni–Sn intermetallic compounds and their packaging solder joints, According to the calculations, Ni_3_Sn has the highest Young’s modulus, fracture toughness, and thermal conductivity. Ni/Ni_3_Sn/Ni solder joints have better comprehensive properties because Ni_3_Sn can match Ni well in the modulus and thermal expansion coefficient, and are suitable for connecting materials of high-temperature power devices. Therefore, the possibility of improving the strength, plasticity, and fracture toughness of the Ni_3_Sn phase is discussed in this paper.

Alloying is an effective way to reduce the brittleness of intermetallic compounds. For example, Yang et al. [10] proved through the density functional theory (DFT) simulation and experiment that the bonding strength between Cu, Ni, and Sn atoms of Cu_6_Sn_5_ and Cu_3_Sn and the stability of the phase structure can be improved by occupying Cu atom sites after adding the Ni element. F. Emadi et al. [11] pointed out that Co participation in the Cu–Sn system can reduce the brittleness of IMCs in the Cu–Sn system. The research on improving the embrittlement of intermetallic compounds in the Ni–Sn system is not deep enough. Co, Cu, and Pt are usually added as additive elements in package solder. Therefore, some others have studied the influence of these elements on the mechanical properties of Ni_3_Sn_4_ [12]. However, the literature only calculates the stability, elastic modulus, and anisotropic characteristics of the phase structure after the addition of alloying elements, not the influence mechanism of alloying elements on the fracture toughness of intermetallic compounds in the Ni–Sn system. Specially, the stress–strain behavior of intermetallic compounds in the Ni–Sn system after alloying under uniaxial tension is not involved. Therefore, it is necessary that we clarify the mechanism of the influence of alloying elements on the mechanical properties of Ni–Sn intermetallic compounds.

In this paper, based on the previous works, the effects of Co, Cu, and Pt on the crystallographic characteristics, structural stability, fracture toughness, and stress–strain behavior of the Ni_3_Sn phase are studied by simulation calculation, thus providing the necessary theoretical support for improving the comprehensive mechanical properties of the Ni_3_Sn phase.

## 2. Calculation Method and Structural Modeling

### 2.1. Calculation Methods

All calculations in this paper were performed using the Vienna ab initio Simulation Package (VASP) code [13]. Projector Augmented Wave Potentials (PAW) [14] and Perdew–Burke–Eruzerhof (PBE) under Generalized Gradient Approximation (GGA) are used to deal with the exchange-correlation energy [15]. By a convergence test, the cut-off energy is set to 440 eV, and the irreducible Brillouin zone is sampled by an 8 × 8 × 8 K-point grid. The self-consistent convergence of the total energy is set at 10^−8^ eV/atom, and the maximum force on the atom is 0.001 eV/Å. Ab initio molecular dynamics (AIMD) simulations were performed using the NVT ensemble [16] to assess thermal stability with a simulation time of 10 Ps. The phonon spectra were calculated using density functional perturbation theory (DFPT) [17] in PHONOPY code [18], using a 3 × 3 × 3 supercell, self-consistent convergence of the total energy set to 10^−8^ eV/atom, and a maximum force on the atom of 1 × 10^−5^ eV/Å.

### 2.2. Structural Modeling

**Figure 1:**
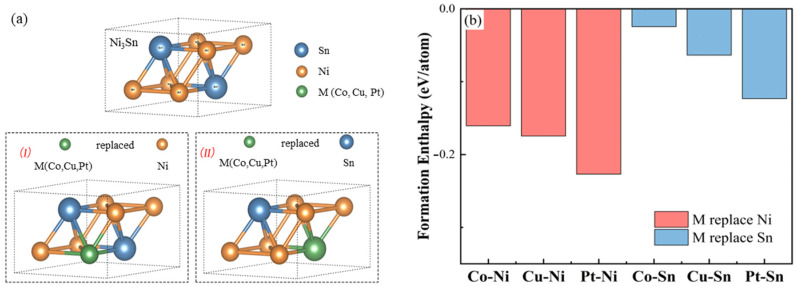
M element occupation model and formation energy of different occupations: (**a**) occupying model; and (**b**) formation energy.

According to the ISCD crystallographic database, among the three intermetallic compounds of the Ni–Sn system, the Ni_3_Sn phase is an arranged hexagonal structure composed of eight atoms. Its Pearson symbol is hP8, and its crystal structure is shown in Figure 1a.

Before constructing the alloy element occupancy model, it is necessary that we first consider the kinds of elements replaced by the Co, Cu, and Pt alloy elements (hereinafter denoted by M). Alloy elements may replace Ni atoms or Sn atoms. Since Ni atoms and Sn atoms occupy only one kind of site in Ni_3_Sn crystal, the atoms of alloying element M replace Ni atoms or Sn atoms and correspond to only one kind of crystal structure, as shown in Figure 1a. In this paper, after M atoms occupy the Ni or Sn atomic sites, respectively, the formation energies of the corresponding structures are calculated as follows:

The equation for calculating the structure formation energy after the M element occupies the Ni atomic site is as follows [19]:

(1) $$E_{formation}^{Ni-\mathrm{site}}=E_{Ni_{5}M_{1}Sn_{2}}-5E_{Ni}-E_{M}-2E_{Sn}$$

The equation for calculating the structure formation energy after the M element occupies the Sn atomic site is as follows:

(2) $$E_{formation}^{Sn-site}=E_{Ni_{6}M_{1}Sn_{1}}-6E_{Ni}-E_{M}-E_{Sn}$$

where $E_{{Ni}_{5}M_{1}{Sn}_{2}}$ and $E_{{Ni}_{6}M_{1}{Sn}_{1}}$ are the total energies of Ni_5_M_1_Sn_2_ and Ni_6_M_1_Sn_1_ crystals after one Ni or Sn atom is replaced by M atom, and $E_{Ni}$, $E_{M}$, and $E_{Sn}$ are the energies of individual Ni, M, and Sn atoms in Ni, M, and Sn crystals.

When Ni or Sn atoms are replaced by alloying element M, the formation energy calculation results of the corresponding structure are shown in Figure 1b. As can be seen from Figure 1b, even when Ni atoms or Sn atoms are replaced by M-type atoms, the formation energy of the corresponding structure is negative. However, the formation energy of the system after M atoms replace Ni atoms is lower, which indicates that M atoms tend to replace the lattice sites of Ni atoms more [20].

**Figure 2:**
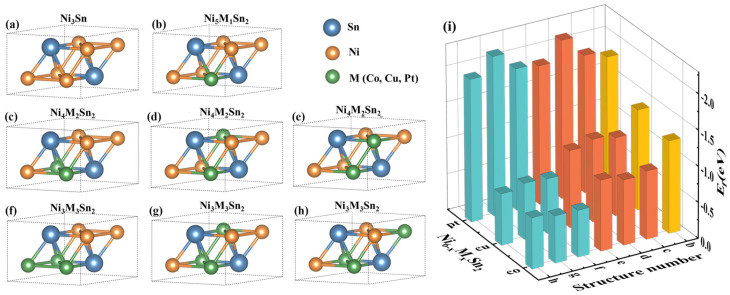
Crystal structure and formation energy of the corresponding structure before and after the addition of alloying elements: (**a**) Ni_3_Sn; (**b**) Ni_5_M_1_Sn_2_; (**c**–**e**) Ni_4_M_2_Sn_2_; (**f**–**h**) Ni_3_M_3_Sn_2_; and (**i**) formation energies of the corresponding structures.

Since Ni occupies only one site in the Ni_3_Sn crystal, there is only one crystal structure for Ni_5_M_1_Sn_2_, as shown in Figure 2b. However, for Ni_4_M_2_Sn_2_ and Ni_3_M_3_Sn_2_ with a high M content, different relative positions of M atoms will form various types of crystal structures. All possible Ni_4_M_2_Sn_2_ and Ni_3_M_3_Sn_2_ phase structures have been considered by permutations and combinations taking into account periodicity, as shown in Figure 2c–h. For example, in Ni_4_M_2_Sn_2_, two M atoms may occupy Ni atoms on the same (0001) plane at the same time, as shown in Figure 2c. It is also possible to occupy Ni atoms located on different (0001) planes, as shown in Figure 2d,e. The difference between (d) and (e) is that the M atoms in (d) are located at two different tetrahedral vertices, whereas the M atoms in (e) are located at the same tetrahedral vertex.

In this paper, the formation energies of all the structures listed in Figure 2b–h are calculated to compare the relative stability of different structures. The formation energies of Ni_6−x_M_x_Sn_2_ are calculated as follows:

(3) $$E_{formation}=E_{{Ni}_{6-x}M_{x}{Sn}_{2}}-\left(6-x\right)E_{Ni}-{xE}_{M}-2E_{Sn}$$

In Equation (3), $E_{{Ni}_{6-x}M_{x}{Sn}_{2}}$ is the energy of Ni_6−x_M_x_Sn_2_ crystal, and $E_{Ni}$, $E_{M}$, and $E_{Sn}$ are the energies of single Ni, M, and Sn atoms in Ni, M, and Sn single crystals, respectively.

The calculated Ni_6−x_M_x_Sn_2_ phase formation energies for all structures are shown in Figure 2i. As can be seen from Figure 2i, when Cu or Pt atoms replace two and three Ni atoms, the structures (d) and (g) in Figure 2 are more likely to form, while Co atoms are more likely to form the structures (e) and (h), which may be due to the differences in atomic radii and interactions between atoms. Therefore, the most stable Ni_6−x_M_x_Sn_2_ phase structure is selected for subsequent calculations.

## 3. Results and Discussion

### 3.1. Crystal Structure and Stability

After determining the structural model of the Ni_3_Sn phase added with the alloying element, the lattice parameters, cell volume, density, and formation energy of the Ni_6−x_M_x_Sn_2_ phase added with the M element were calculated, and listed in Table 1. It can be seen from Table 1 that, for Ni_6−x_M_x_Sn_2_ (M is Cu and Pt, x = 1~3), the volume after optimization increases, and increases with the increase in alloying element concentration, while the addition of the Co element shows the opposite trend. Among all M elements, the Pt element has the greatest influence on the volume change of the Ni_3_Sn phase, which may be related to the atomic radius of alloying elements.

Volume shrinkage porosity is a common cause of solder joint defects in the TLPB process. When IMCs are formed from matrix elements, the crystal structure and density change. In general, the formation of IMCs is accompanied by local volume shrinkage, and tensile stress is generated in the overall package solder joint due to the structural constraints around IMCs. Therefore, the solder joint must have a certain plasticity, so that, once the package stress exceeds the yield strength, the tensile stress caused by volume shrinkage is released by plastic deformation to prevent the cracking of the solder joint. From the crystal structure characteristics of typical metals, the densities of FCC and HCP crystal materials are similar, about 0.74, so pure Ni, pure Sn, and HCP Ni_3_Sn phases of the FCC structure have similar densities at room temperature. However, Sn is a liquid state in TLPB, the atomic distance becomes larger, and the density is slightly lower than that of solid-state Sn at room temperature. Therefore, the phase transition reaction process Ni (FCC) + Sn (Liquid) → Ni_3_Sn (HCP) may be density-increasing. Therefore, it is probable that volume shrinkage may occur during the Ni + Sn → Ni_3_Sn reaction. The theoretical molar volume shrinkage of Ni + Sn → Ni_3_Sn and the effect of alloying elements on the molar volume shrinkage are estimated from the phase transformation reaction equation below.

The formation reaction Equation (4) of Ni_3_Sn is as follows:

(4) $$6Ni+2Sn(L)\to 2Ni_{3}Sn$$

The volume change before and after the reaction can be calculated from Equation (5):

(5) $$\Delta v=2v_{Ni_{3}Sn}-6v_{Ni}-2v_{Sn}$$

The volume change rate η can be calculated from Equation (6):

(6) $$\eta =\frac{\Delta \upsilon }{v}=\frac{2v_{Ni_{3}Sn}-6v_{Ni}-2v_{Sn}}{6v_{Ni}+2v_{Sn}}$$

Similarly, after the addition of alloying elements, the volume change rate η of Ni_6−x_M_x_Sn_2_ can be calculated from Equation (7):

(7) $$\eta =\frac{\Delta \upsilon }{v}=\frac{v_{Ni_{6-x}M_{x}{Sn}_{2}}-(6-x)v_{Ni}-xv_{M}-2v_{Sn}}{(6-x)v_{Ni}+xv_{M}+2v_{Sn}}$$

Further expressing the molar volume in terms of molar mass and density, the rate of change per molar volume can be expressed as follows:

(8) $$\eta =\frac{\Delta \upsilon }{v}=\frac{M_{Ni_{6-x}M_{x}{Sn}_{2}}/\rho_{Ni_{6-x}M_{x}{Sn}_{2}}-(6-x)M_{Ni}/\rho_{Ni}-xM_{M}/\rho_{M}-2M_{Sn}/\rho_{Sn}}{(6-x)M_{Ni}/\rho_{Ni}+xM_{M}/\rho_{M}+2M_{Sn}/\rho_{Sn}}$$

In Equations (7) and (8), M is the molar mass and ρ is the density.

**Figure 3:**
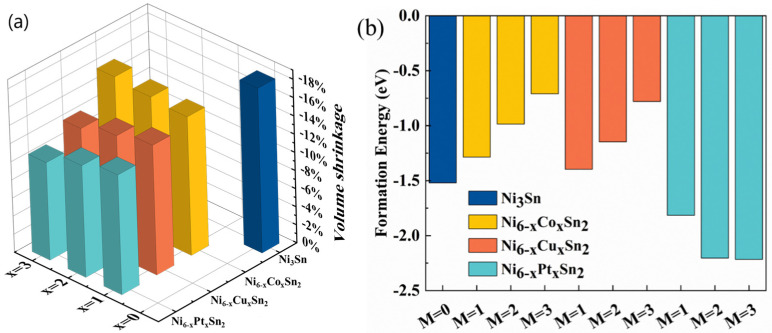
The volume shrinkage and formation energy of Ni_3_Sn or Ni_6−x_M_x_Sn_2_ phases before and after the addition of alloying elements: (**a**) volume shrinkage; and (**b**) formation energy.

Since Sn exists in liquid form in TLPB and the TLPB process temperature is usually 300 °C, the density of Sn used in the above calculation is 6.934 g/cm^3^ (300 °C) [21]. The calculation results are shown in Figure 3a. It can be seen from the figure that Ni_3_Sn generated from Ni and Sn will undergo an 18% volume shrinkage, which will generate large local tensile stress in the package solder joint, which may lead to the early fracture failure of the solder joint.

When M is added, the theoretical molar volume shrinkage can be reduced by about 2–7% when the Ni_6−x_M_x_Sn_2_ phase is formed with Ni and Sn. If the solder joint is composed of a single dense Ni_6−x_M_x_Sn_2_ phase by adjusting the welding process, the local tensile stress caused by shrinkage can be effectively reduced, and the shrinkage pores in the solder joint can be reduced. In addition, for the Ni_6−x_M_x_Sn_2_ phase with a Cu and Pt addition, the volume shrinkage will further decrease with the increase in alloying element content, thus reducing the local tensile stress concentration more effectively. When x = 3, the volume shrinkage of the Ni_3_Pt_3_Sn_2_ phase formed by Ni, Pt, and Sn elements is 11%, which is 7% lower than that without the addition of alloying elements.

Table 1 lists the lattice parameters, unit cell volume, density, and formation energy of the Ni_3_Sn phase before and after alloying.

From the results of the formation energy of each IMCs in Table 1, it can be seen that the formation energy of all IMCs is negative, which indicates that all the above structures have thermal stability. The formation energy of the Ni_6−x_M_x_Sn_2_ phase decreases with the Pt addition and increases with the Co and Cu addition. It can be seen that the Ni_6−x_Pt_x_Sn_2_ phase is easier to form and has higher thermal stability than the pure Ni_3_Sn phase.

**Figure 4:**
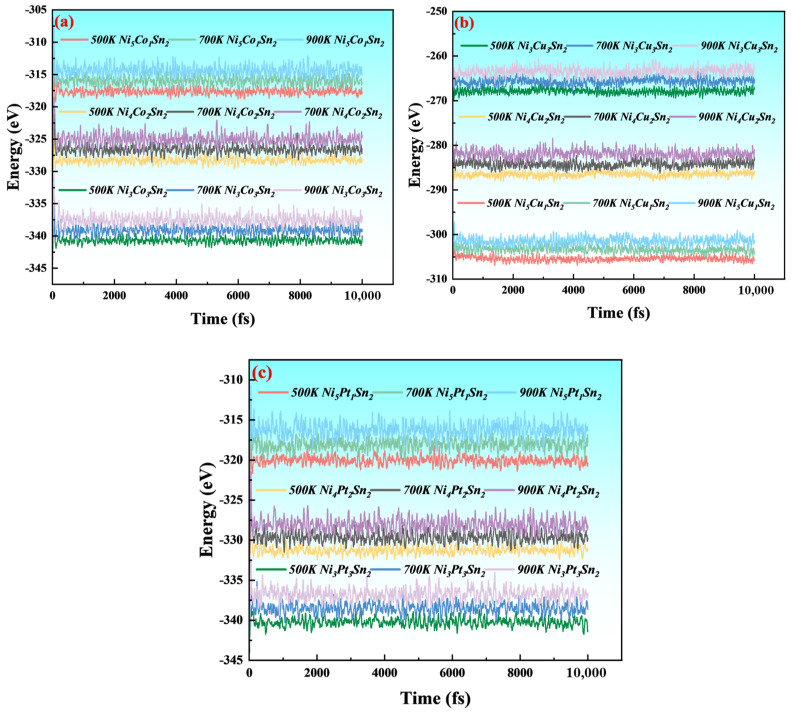
AIMD measurement (500~900 K, 10 Ps) of the total energy change of Ni_6−x_M_x_Sn_2_ system: (**a**) Ni_6−x_Co_x_Sn_2_; (**b**) Ni_6−x_Cu_x_Sn_2_; and (**c**) Ni_6−x_Pt_x_Sn_2_.

In order to confirm the thermal stability of the Ni_6−x_M_x_Sn_2_ phase, ab initio molecular dynamics (AIMD) tests were performed on the Ni_6−x_M_x_Sn_2_ phase. As shown in Figure 4, Ni_6−x_M_x_Sn_2_ was heated for 10 Ps at temperatures of 500 K, 700 K, and 900 K, and the total energy of the Ni_6−x_M_x_Sn_2_ system fluctuated within a small range over time. In addition, the model structure of the Ni_6−x_M_x_Sn_2_ phase has no significant change before and after the test, and the bonding bond has not broken, which indicates that the Ni_6−X_M_X_Sn_2_ phase structure constructed in this paper has a good thermal stability [22,23].

**Figure 5:**
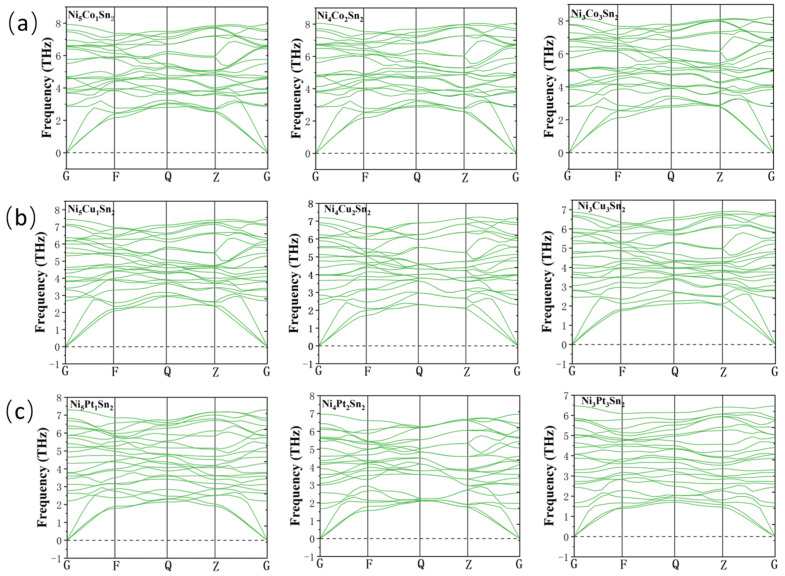
Phonon spectrum of Ni_6−x_M_x_Sn_2_: (**a**) Ni_6−x_Co_x_Sn_2_; (**b**) Ni_6−x_Cu_x_Sn_2_; and (**c**) Ni_6−x_Pt_x_Sn_2_.

In addition, stable crystal structures require that all phonon frequencies must not be negative. In this paper, the phonon spectra of the corresponding structure with alloying elements added are calculated according to the density functional perturbation theory (DFPT). The results are shown in Figure 5.

It can be seen from Figure 5 that the phonon frequency in any direction is greater than 0, so the structure is dynamically stable after the addition of alloying elements [24,25].

### 3.2. Effect of Alloying Elements on Mechanical Properties of Ni_3_Sn

In order to study the mechanical properties of the Ni_6−x_M_x_Sn_2_ crystal structure, the elastic constants ($C_{ij}$) of the Ni_6−x_M_x_Sn_2_ crystal were calculated by the stress–strain method. The shear modulus (G), bulk modulus (B), Young’s modulus (E), and Poisson’s ratio (ν) of the material are obtained by using Voigt–Reuss–Hill (VRH) approximation [26,27,28]. The elastic constants C_ij_ obtained by the stress–strain method are listed in Table 2. The values of G, B, E, and ν obtained by VRH approximation are listed in Table 3.

The mechanical stability of Ni_3_Sn doped with alloying elements was evaluated by the Born–Huang crystal mechanical stability criterion. For close-packed hexagonal structures, the mechanical stability of crystals can be evaluated by elastic constants [29]: $C_{44}>0,\left(C_{11}+C_{12}\right)C_{33}-2C_{13}^{2}>0,C_{11}-|C_{12}|>0$. The calculation results show that all structures satisfy the Bonn crystal mechanical stability criterion, indicating that the Ni_3_Sn phase has mechanical stability regardless of the alloying elements. Based on the calculation results of the formation energy, AIMD, phonon spectrum, and Born–Huang crystal stability criterion, the phase structures of all models established in this paper are stable.

As can be seen from Table 2, the addition of Cu and Pt elements decreases the elastic constants of the Ni_3_Sn phase in all directions, and the elastic constants decrease continuously with the increase in Cu and Pt. This indicates that Cu and Pt elements can reduce the ability of the Ni_3_Sn phase to resist compression and shear deformation, and also reflects that Cu and Pt elements can weaken the bonding strength between atoms in the crystal. Co increases the elastic constants in all directions and increases with the increase in element content. Therefore, it can be predicted that the bonding strength between atoms in the Ni_6−x_Co_x_Sn_2_ system is the strongest, and, therefore, the ability to resist elastic deformation caused by service load is also the strongest. In addition, with the increase in Co content, the values of C_44_ and C_66_ are closer and this indicates that the shear anisotropy becomes smaller and smaller.

**Figure 6:**
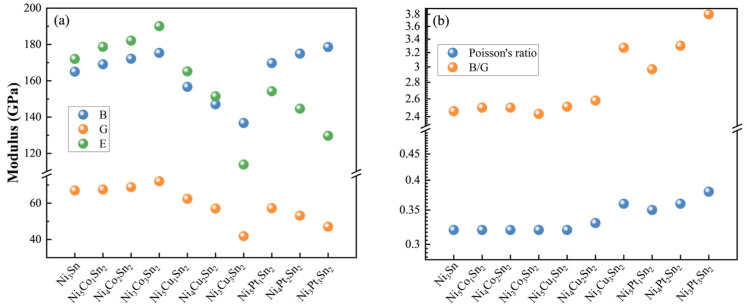
Changes in modulus, B/G, and Poisson’s ratio before and after alloying elements addition: (**a**) bulk modulus (B), shear modulus (G), and Young’s modulus (E); and (**b**) Pugh ratio (B/G), and Poisson’s ratio (ν).

The bulk modulus is a measure of the volume change of a material under uniform compression, while the shear modulus expresses how difficult is for a material to shear under stress. It can be seen from Figure 6a that the addition of the Co will increase the modulus of the system, while the addition of Cu and Pt reduce the modulus of the system to a certain extent, especially the shear modulus.

It is pointed out in the literature that the Pugh ratio (B/G) and Poisson ratio ν can reflect the toughness and brittleness of the materials [30,31,32,33]. The larger the B/G value and Poisson ratio, the better the plasticity. The B/G values of the system are all greater than 1.75 after the alloying elements are added, which can be regarded as ductile materials according to the theory. It can be seen from Figure 6b that alloying elements have different degrees of influence on the B/G value of Ni_3_Sn, among which Pt is the most significant element. The Pt addition can increase the B/G ratio and Poisson ratio of Ni_3_Sn. According to this criterion, it can be inferred that the brittleness of the Ni_3_Sn phase can be reduced remarkably by adding the Pt element.

In order to further reveal the comprehensive effect of alloying elements on the strength and toughness of Ni_3_Sn, the tensile tests of Ni_6−x_M_x_Sn_2_ were carried out according to the first principles. According to the Nielsen–Martin principle [34], the theoretical tensile strength is calculated by gradually applying strain ε, and the relevant calculation formulae are shown in Equations (9) and (10).

(9) $$\varepsilon =\left(l_{n}-l_{0}\right)/l_{0}\times 100\%$$

(10) $$\sigma =\frac{1}{\Omega }\frac{\partial E_{e}}{\partial \varepsilon }$$

where $l_{n}$ is the lattice length after *n* times of strain, $l_{0}$ is the initial lattice length, σ is the stress, $E_{e}$ is the total energy of the system, and Ω is the unit cell volume.

In this paper, the tensile step size is 0.02, the tensile direction is perpendicular to the (0001) crystal plane along the high symmetry crystal direction [0001], and the tensile stops when the strain reaches 26%. The Poisson’s ratio effect is considered in the calculation, and the lattice constant is optimized.

**Figure 7:**
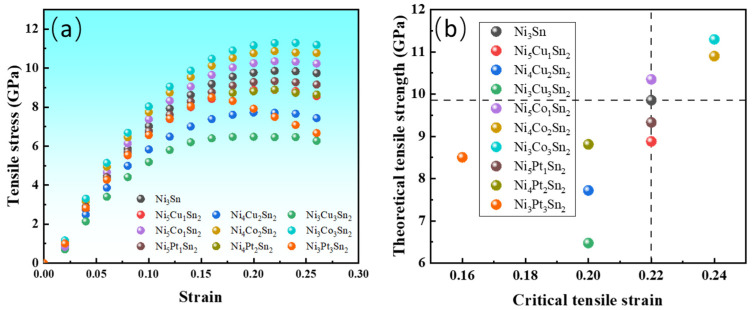
The tensile stress–strain curves and critical strain–tensile strength distribution along the [0001] direction before and after the addition of alloying elements: (**a**) tensile stress–strain curve; and (**b**) critical strain and tensile strength distribution.

If the corresponding strain to the tensile strength of the applied stress is called a critical strain, for brittle materials, when the critical strain is exceeded, the local bond in the material may fracture, and the tensile strength is close to the fracture strength. The corresponding critical strain can indirectly characterize its fracture resistance. For materials with good plasticity, since the degree of elastic deformation is much smaller than plastic deformation, the critical strain can also approximately reflect the plasticity of the material [24]. It can be seen from the simulated tensile results of Figure 7a,b that the Ni_6−x_M_x_Sn_2_ material system has a certain plasticity.

When the content of alloy elements is low, the influence on the critical strain is small. Cu and Pt decrease the critical strain with the increase in alloying element content, which indicates that Cu and Pt decrease the plasticity of the Ni_3_Sn phase, while Co increases the plasticity and tensile strength. The area contained in the stress–strain curve under uniaxial tension represents the static toughness (toughness) of the material. When the tensile curves are integrated from 0 to the critical strain value, it is found that the order of integrated area is Ni_6−x_Co_x_Sn_2_ > Ni_3_Sn > Ni_6−x_Pt_x_Sn_2_ > Ni_6−x_Cu_x_Sn_2_ when the x values are equal. The calculation results show that Cu and Pt decrease the toughness of Ni_3_Sn, while the Co can increase the toughness.

The fracture toughness of Ni_6−x_M_x_Sn_2_ was calculated using Niu et al.’s empirical fracture toughness model (Equation (11)) [35].

(11) $$K_{\mathrm{IC}}{=V}_{0}^{1/6}{G(B/G)}^{1/2}$$

where $V_{0}$ denotes the volume of a single atom, and B and G denote the bulk modulus and shear modulus, respectively.

The relevant calculation results are shown in Table 3. The fracture toughness range of Ni_6−x_M_x_Sn_2_ is 1.4~1.8 Mpa·m^1/2^. When x is equal, the order of fracture toughness is Ni_6−x_Co_x_Sn_2_> Ni_3_Sn > Ni_6−x_Pt_x_Sn_2_> Ni_6−x_Cu_x_Sn_2_. The smaller the fracture toughness, the lower the ability to prevent crack instability propagation, which indicates that Cu and Pt will increase the brittleness of Ni_3_Sn, which is consistent with the results obtained from the tensile simulation. From the results obtained from the tensile simulation and fracture toughness, it is found that the addition of Co is favorable for improving the brittleness of the Ni_3_Sn phase. In the foregoing, it is inferred that the addition of the Pt is beneficial in improving the brittleness of the Ni_3_Sn phase by the B/G criterion, and there is a discrepancy between the two. In Section 4, the reasons for these differences between the two criteria are discussed.

### 3.3. Effect of Alloying Elements on Ni_3_Sn Anisotropy

Elastic anisotropy can be reflected by the general anisotropy index (A_U_), bulk modulus anisotropy index (A_B_), and shear modulus anisotropy index (A_G_), which can be calculated by the following equations [36], respectively:

(12) $$A_{U}=5\frac{G_{V}}{G_{R}}+\frac{B_{V}}{B_{R}}-6$$

(13) $$A_{B}=\frac{B_{V}-B_{R}}{B_{V}+B_{R}}\times 100\%$$

(14) $$A_{G}=\frac{G_{V}-G_{R}}{G_{V}+G_{R}}\times 100\%$$

where G_V_, B_V_, G_R,_ and B_R_ represent the approximate values of the moduli simulated by the Voigt and Reuss functions, respectively.

**Figure 8:**
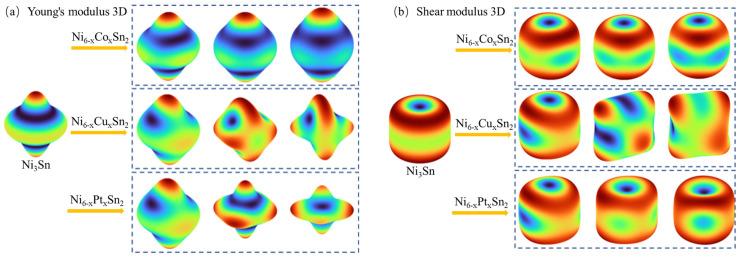

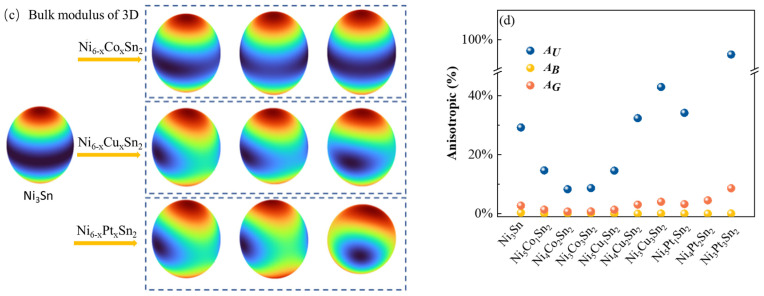
The three-dimensional structure and anisotropy index of the modulus of Ni_6−x_M_x_Sn_2_: (**a**) Young’s modulus; (**b**) shear modulus; (**c**) bulk modulus; and (**d**) anisotropy index.

In addition, the detailed information on the elastic three-dimensional orientation distribution of the Ni_6−x_M_x_Sn_2_ crystal is described intuitively according to the ELAM code [37]. As can be seen from Figure 8a–d, the three-dimensional diagram of the modulus of the crystal after the alloy element replaces Ni atoms has different changes. The Co element can reduce the deviation of the three-dimensional modulus diagram from the sphere, and the anisotropy index also decreases, while the Cu and Pt elements are the opposite. This indicates that Co can decrease the anisotropy of the Ni_3_Sn phase, while Cu and Pt can increase the anisotropy of the Ni_3_Sn phase, and the anisotropy becomes larger with the increase in Co content. Among them, the Ni_6−x_Pt_x_Sn_2_ phase has the largest difference in mechanical properties in different directions, which makes it easy to induce microcracks due to the uneven stress field during service load, thus reducing the strength of solder joints.

### 3.4. Effect of Alloying Elements on the Electronic Structure of Ni_3_Sn

**Figure 9:**
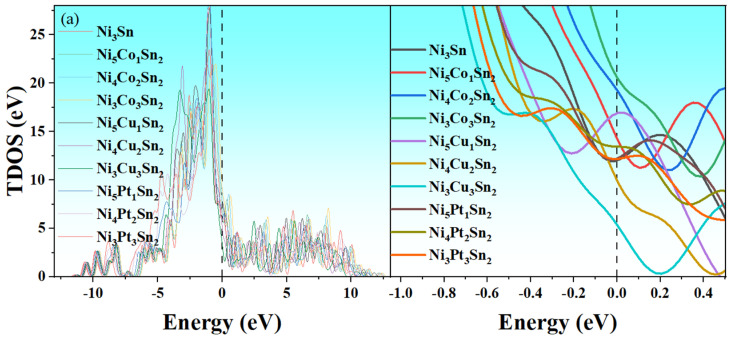

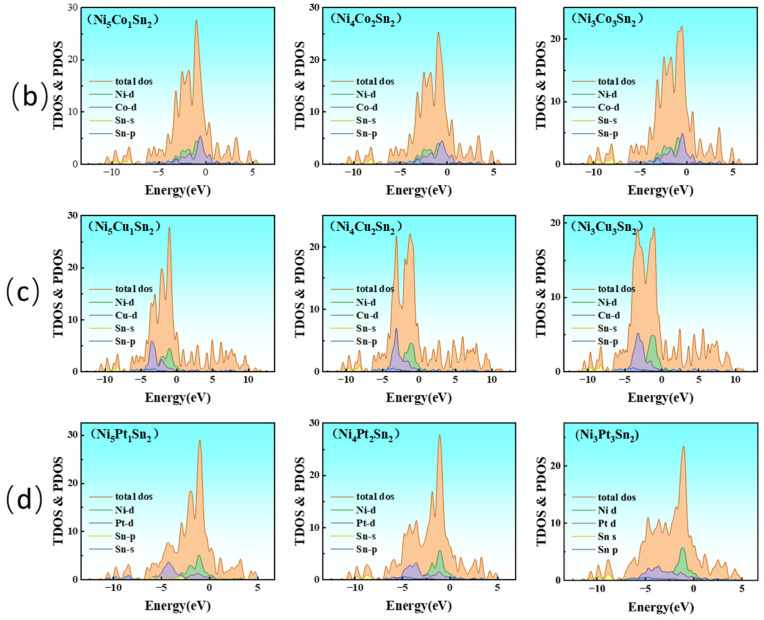
Total density of states and partial density of states of Ni_3_Sn phase before and after adding alloying elements. (**a**) TDOS; (**b**) DOS of Ni_6−x_Co_x_Sn_2_; (**c**) DOS of Ni_6−x_Cu_x_Sn_2_; (**d**) DOS of Ni_6−x_Pt_x_Sn_2_.

The electronic structure significantly affects the mechanical properties of Ni_3_Sn intermetallic compounds, affecting the bonding characteristics between the constituent elements. In order to further explore the effect of alloying elements on the bonding properties of Ni_3_Sn intermetallic compounds, the total density of states (TDOS) and the partial density of states (PDOS) of the Ni_3_Sn phase before and after alloying elements are calculated. The calculation results are shown in Figure 9. Because the electron orbitals of transition elements are mainly contributed by d orbitals, the density curves of states for the d orbitals of Ni, Co, Cu, and Pt are plotted.

It can be seen from Figure 9a that the density of states of Ni_3_Sn is mainly distributed in the energy range of 11.0 eV to 10 eV, and the TDOS distribution changes when alloying elements are added. The energy distribution range of TDOS will shift to a lower energy state with a Pt addition, and the shift amplitude will be larger with the increase in Pt content. These results indicate that the stability of the Ni_3_Sn phase can be enhanced by a Pt addition, and the stability increases with the increase in Pt content, which is consistent with the conclusion of the formation energy.

Whether or not alloy elements are added, the system has a large TDOS value at the Fermi level; that is, the TDOS value is not 0, indicating that Ni_6−x_M_x_Sn_2_ is a conductor and exhibits typical metallic characteristics. The bonding properties of metals are proportional to the TDOS value at the Fermi level (E_f_) [38]. It can be seen from Figure 9a that the TDOS value at E_f_ changes little when the alloy element content is low. Ni_3_Co_3_Sn_2_ has a maximum TDOS value of 8.9 eV at E_f_. Ni_3_Cu_3_Sn_2_ has the lowest TDOS value at E_f_, about 3.5 eV. This indicates that Co can enhance the metal bonding properties of Ni_3_Sn, while Cu can weaken them.

The curves of the density states are shown in Figure 9b–d. It can be seen from the figure that, for the system with Co addition, TDOS is mainly contributed by Ni-d and Co-d electrons, and the three main bonding peaks are located between 4 eV and 2 eV. Ni-d orbitals and Co-d orbitals produce hybridization, forming strong bonding interactions. For the Cu-doped system, there are two main bonding peaks located at 2.2 eV to 1.7 eV and 3.5 eV to 2.8 eV when the Cu content is low, which are generated by the hybridization of Ni-d orbitals and Cu-d orbitals. With the increase in Cu content, the bonding peak at −3.5 eV to −2.8 eV decreases obviously, and the bonding peak at −2.2 eV to −1.7 eV shifts to the right. Finally, the bonding peak at −3.5 eV to −2.8 eV disappeared. The above phenomena indicate that the bonding effect of the Ni–Cu bond becomes weaker with the increase in Cu content. For the system with a Pt addition, similar to the system containing Cu, the bonding effect becomes less and less obvious with the increase in Pt content. These results indicate that Co can increase the bonding strength of the system and, thus, improve strength, while Cu and Pt can weaken the bonding strength of the system and cause the strength to decrease.

The Bader charge analysis quantifies the transfer and distribution of local atomic charges by partitioning the electron density space, providing direct evidence for revealing the evolution of chemical bond characteristics (such as metallicity, covalency, or ionicity) during the alloying process. Table 4 summarizes the average net charges of Ni, Sn, and alloying elements in different alloy systems, intuitively reflecting the charge regulation mechanism of each element at the electronic level.

The net charge of Sn without an alloying element addition is +0.46, while the net charge of Ni is −0.15 to −0.16. This indicates that Sn acts as an electron donor, losing partial electrons, while Ni serves as an electron acceptor, forming stable metal–covalent hybrid bonds with Sn. After the Co addition, the charge of Co approaches neutrality, suggesting minimal electron transfer with Ni/Sn and maintaining metallic bond characteristics. The net charge of Sn, +0.43 to +0.45, shows a slightly reduced electron loss, likely due to Co optimizing the charge distribution through d-orbital hybridization. The net charge of Ni, −0.15 to −0.17, remains essentially unchanged, indicating that the Co addition does not significantly disrupt the original bonding states. After the Cu addition, Cu’s charge approaches neutrality but partial electrons may transfer to Sn through localized hybridization. The net charge of Sn, +0.34 to +0.38, reflects a reduced electron loss, suggesting Cu alleviates Sn’s charge transfer. The net charge of Ni, −0.17 to −0.20, indicates an increased electron gain, possibly due to Cu altering the bonding states through electron sharing. After the Pt addition, Pt’s net charge, −0.56 to −0.79, demonstrates a strong electron attraction, forming localized charge centers. The net charge of Sn, +0.70 to +0.82 (compared to the original +0.46), shows significant electron loss due to Pt’s high electronegativity. The net charge of Ni, +0.02 to +0.13, indicates the charge reversal from electron acceptor to donor caused by a localized charge imbalance, disrupting the original bonding. In summary, (1) Co slightly alleviates Sn’s electron loss through d-orbital hybridization, enhancing the bond strength and improving elasticity and toughness; (2) Cu weakens the polar bonds between Sn and Ni, with the weaker Cu-d/Ni-d hybridization reducing the overall mechanical properties; and (3) Pt’s strong localized charge causes severe Sn electron loss and Ni charge reversal, leading to embrittlement.

## 4. Discussion

According to the theoretical estimation of the equation of the phase transformation reaction of alloy formation, the volume shrinkage rate of Ni + Sn → Ni_3_Sn is about 18%. If the addition of alloying elements can produce a single dense Ni_6−x_M_x_Sn_2_ phase, this volume shrinkage will be reduced, among which the Pt element is the most effective, Cu element is the second, and Co is the last. Therefore, it may be a new way to alleviate the volume shrinkage of the Ni–Sn system by adding alloying elements during the preparation process of TLPB.

The results show that the Pugh ratio (B/G) of Ni_6−x_M_x_Sn_2_ (M = Cu, Pt) is much larger than 1.75. According to the B/G criterion, Ni_6−x_M_x_Sn_2_ (M = Cu, Pt) should have good plasticity, but the simulated tensile stress–strain curves do not show that Ni_6−x_M_x_Sn_2_ (M = Cu, Pt) has good plasticity. Furthermore, the classical toughness and brittleness criterion of B/G indicates that Pt can improve the brittleness of the Ni_3_Sn phase more obviously. The results of the tensile and fracture toughness calculation based on the first-principles simulation show that the brittleness of the Ni_3_Sn phase is improved more effectively after the Co addition, and the conclusions obtained by the B/G classical toughness criterion are different from those obtained by the first-principles simulation. The above situation is also reflected in the relevant literature; for example, the Pugh ratio (B/G) of the NiA1 alloy phase is calculated to be 2.26, and, according to the B/G classical ductile–brittle criterion, NiA1 should have ductility, but, in fact, the NiA1 alloy phase shows brittleness [39]. The B/G ratio of NbCr_2_ is 3.69, but the fracture toughness is about 1~1.4 MPa·m^1/2^, and a large number of experiments show that NbCr_2_ is also brittle [40].

According to Pugh et al. [30], the research system is pure metal, the crystal structure is usually simple, and the bonding type is single, but the bonds of intermetallic compounds are generally a mixture of metallic, ionic, and covalent bonds. In addition, it is mentioned in the Pugh literature that lattice structures and slip systems, especially hexagonal structures, must be considered when predicting the plasticity of materials. This is reflected in the study of Xing et al. [39]. The energy of NiA1 antiphase boundaries (APBs) is high, and there is a lack of a sufficient slip system, so the ductility is poor. The systems studied in this paper are complex intermetallics with an HCP structure, which lack slip systems and have complex bonding types, which is why that the B/G criterion is not applicable. Finally, Pugh et al. published, in 1954, a study on the evaluation of the plastic deformation characteristics of materials by the B/G criterion [30], which is based on the energy theory of edge dislocation and screw dislocation. In a large number of subsequent studies on the microscopic mechanism of the plastic deformation of materials, it was found that the dislocation morphology in the plastic deformation process of actual engineering materials was more complex than the original simplified assumption of single-edge dislocation and screw dislocation [41,42]. Therefore, the influence mechanism of the elastic stress field based on the energy of simple edge dislocation and screw dislocation on the plastic deformation of materials (characterized by yield strength, tensile strength, and plasticity) had certain limitations. However, the empirical model of fracture toughness of Niu et al. [35] fits the mixed-bond crystal, with a correlation coefficient of 0.97 and a root mean square error of 0.4MPa·m^1/2^, which has a high reliability. Therefore, it is considered that the fracture toughness *K*_IC_ may be more appropriate for evaluating the toughness of the system materials.

## 5. Conclusions

The lattice parameters, cell volume, density, and formation energy of the Ni–Sn system with alloying elements were calculated. An occupancy model of Co, Cu, and Pt in the Ni_3_Sn intermetallic compound phase was constructed. The effects of Co, Cu, and Pt on the mechanical properties of Ni_3_Sn intermetallic compounds were studied using the first-principles calculation, Pugh ratio (B/G), fracture toughness *K*_IC_ calculation, and stress–strain curve analysis of simulated tension. The main conclusions are as follows:

(1) The formation energy calculation and electronic structure analysis show that the Ni_6−X_M_X_Sn_2_ phase structure with Co, Cu, and Pt additions has good thermal stability.
(2) Co, Cu, and Pt can reduce the theoretical volume shrinkage of the Ni + Sn → Ni_3_Sn reaction, and Pt can reduce the volume shrinkage by about 7%.
(3) With the increase in alloy element content, Cu and Pt will increase the anisotropy of the Ni_3_Sn phase, and the addition of Cu and Pt will easily lead to an uneven stress field and induce the cracking of intermetallic compound solder joints during service.
(4) The first-principles tensile and fracture toughness analysis results show that the toughness of Ni_3_Sn can be improved by adding the Co. Since the formation of Ni_6−x_Co_x_Sn_2_ increases with the increase in Co content, the toughness of the Ni_3_Sn phase can be improved by adding a low Co content.
(5) The B/G criterion is not suitable for evaluating the toughness and brittleness of Ni–Sn–M alloy systems with complex bonding types after Co, Cu, and Pt are added.

## Figures and Tables

**Table 1:** The lattice parameters, volume *V* (Å), density *ρ* (g/m^3^), and formation energy *E_f_* (eV) of Ni_3_Sn phase before and after alloying elements addition.

IMCs	Lattice			V	ρ	$E_{f}$
	a	b	c			
Ni_3_Sn	5.31	5.31	4.26	104.2	9.76	−1.52
Ni_5_Cu_1_Sn_2_	5.36	5.33	4.29	105.8	9.32	−1.40
Ni_4_Cu_2_Sn_2_	5.43	5.33	4.32	107.6	9.24	−1.15
Ni_3_Cu_3_Sn_2_	5.46	5.40	4.34	109.7	9.14	−0.78
Ni_5_Co_1_Sn_2_	5.29	5.33	4.24	103.9	9.42	−1.29
Ni_4_Co_2_Sn_2_	5.31	5.31	4.22	103.5	9.46	−0.98
Ni_3_Co_3_Sn_2_	5.32	5.32	4.20	103.1	9.50	−0.71
Ni_5_Pt_1_Sn_2_	5.31	5.32	4.26	110.1	10.95	−1.82
Ni_4_Pt_2_Sn_2_	5.56	5.45	4.10	114.7	12.47	−2.20
Ni_3_Pt_3_Sn_2_	5.62	5.57	4.47	120.5	13.76	−2.21

**Table 2:** The $C_{ij}$ (GPa) of Ni_6−x_M_x_Sn_2_.

IMCs	*C*_11_	*C*_22_	*C*_33_	*C*_44_	*C*_55_	*C*_66_	*C*_12_	*C*_13_	*C*_23_
Ni_3_Sn	263.9	263.9	289.5	48.4	47.9	73.6	111.7	109.7	110.6
Ni_5_Co_1_Sn_2_	262.9	260.9	298.1	57.4	55.2	72.7	121.3	114.7	114.7
Ni_4_Co_2_Sn_2_	263.3	264.8	301.0	60.1	61.0	69.7	120.4	120.5	120.5
Ni_3_Co_3_Sn_2_	268.1	270.2	315.8	64.3	64.9	70.5	122.9	121.4	119.4
Ni_5_Cu_1_Sn_2_	245.1	239.1	272.2	50.7	57.8	64.6	114.8	105.4	107.1
Ni_4_Cu_2_Sn_2_	228.4	233.1	247.8	43.7	57.8	58.5	102.2	106.6	98.6
Ni_3_Cu_3_Sn_2_	194.6	184.0	219.9	37.6	40.4	45.7	111.6	99.6	105.6
Ni_5_Pt_1_Sn_2_	259.0	260.5	280.2	41.6	42.7	66.0	119.0	125.1	120.4
Ni_4_Pt_2_Sn_2_	262.7	265.6	278.7	37.5	36.9	62.3	125.9	132.1	126.2
Ni_3_Pt_3_Sn_2_	266.1	248.5	277.3	27.7	33.9	66.2	137.3	133.5	137.9

**Table 3:** The modulus (B, G, and E, GPa), Poisson’s ratio (*ν*), Pugh ratio (B/G), and fracture toughness (*K*_IC_, MPa·m^1/2^) of Ni_6−x_M_x_Sn_2_.

IMCs	B	G	E	*ν*	B/G	*K*_IC_
Ni_3_Sn	165.00	67.00	172.00	0.32	2.46	1.61
Ni_5_Co_1_Sn_2_	169.07	67.49	178.70	0.32	2.50	1.64
Ni_4_Co_2_Sn_2_	172.12	68.80	182.13	0.32	2.50	1.67
Ni_3_Co_3_Sn_2_	175.35	72.04	190.08	0.32	2.43	1.72
Ni_5_Cu_1_Sn_2_	156.65	62.36	165.17	0.32	2.51	1.52
Ni_4_Cu_2_Sn_2_	147.03	57.02	151.47	0.33	2.58	1.41
Ni_3_Cu_3_Sn_2_	136.72	41.83	113.87	0.36	3.27	1.17
Ni_5_Pt_1_Sn_2_	169.70	57.19	154.23	0.35	2.97	1.52
Ni_4_Pt_2_Sn_2_	174.96	53.09	144.64	0.36	3.30	1.50
Ni_3_Pt_3_Sn_2_	178.59	47.02	129.68	0.38	3.80	1.44

**Table 4:** Net charge of atoms before and after adding alloying elements.

Net Charge (Average)			
Alloy System	Ni	Sn	Alloying Element (Co/Cu/Pt)
Pure Ni_3_Sn	−0.15	0.46	-
Ni_5_Co_1_Sn_2_	−0.16	0.45	Co: −0.10
Ni_4_Co_2_Sn_2_	−0.16	0.43	Co: −0.11
Ni_3_Co_3_Sn_2_	−0.16	0.42	Co: −0.12
Ni_5_Cu_1_Sn_2_	−0.17	0.42	Cu: −0.01
Ni_4_Cu_2_Sn_2_	−0.18	0.38	Cu: −0.02
Ni_3_Cu_3_Sn_2_	−0.19	0.34	Cu: −0.03
Ni_5_Pt_1_Sn_2_	−0.06	0.56	Pt: −0.79
Ni_4_Pt_2_Sn_2_	0.03	0.7	Pt: −0.76
Ni_3_Pt_3_Sn_2_	0.08	0.81	Pt: −0.63

## Data Availability

The original contributions presented in this study are included in the article. Further inquiries can be directed to the corresponding authors.

## Abbreviations

The following abbreviations are used in this manuscript:

TLPB	transient liquid-phase bonding
IMCs	Intermetallic compounds
DFT	density functional theory
VASP	Vienna ab initio Simulation Package
PAW	Projector Augmented Wave Potentials
PBE	Perdew–Burke–Eruzerhof
GGA	Generalized Gradient Approximation
AIMD	ab initio molecular dynamics
DFPT	density functional perturbation theory
VRH	Voigt–Reuss–Hill
TDOS	total density of states
PDOS	partial density of states
APB	antiphase boundaries

## References

[1] Holaday J.R., Handwerker C.A., Siow K.S. (2019). Transient Liquid Phase Bonding. Die-Attach Materials for High-Temperature Applications in Microelectronics Packaging: Materials, Processes, Equipment, and Reliability.

[2] Cook G.O., Sorensen C.D. (2011). Overview of transient liquid phase and partial transient liquid phase bonding. J. Mater. Sci..

[3] Jung D.H., Sharma A., Mayer M., Jung J.P. (2018). A Review on Recent Advances in Transient Liquid Phase (TLP) Bonding for Thermoelectric Power Module. Rev. Adv. Mater. Sci..

[4] Chen Z., Liu P., Ren J., Huang M. Full Ni_3_Sn_4_ IMC interconnects prepared by current driven bonding (CDB) method. Proceedings of the 2023 24th International Conference on Electronic Packaging Technology (ICEPT).

[5] Murayama K., Higashi M., Sakai T., Imaizumi N. Electro-migration behavior in low temperature flip chip bonding. Proceedings of the 2012 IEEE 62nd Electronic Components and Technology Conference.

[6] Sun F., Pan Z., Liu Y. (2021). The fracture mechanism of Cu3Sn-microporous copper composite joint by thermal compression bonding process. Mater. Lett..

[7] Yu L.J., Yen H.W., Wu J.Y., Yu J.J., Kao C.R. (2017). Micromechanical behavior of single crystalline Ni_3_Sn_4_ in micro joints for chip-stacking applications. Mater. Sci. Eng. A.

[8] Liu L., Chen Z., Liu C., Wu Y., An B. (2016). Micro-mechanical and fracture characteristics of Cu_6_Sn_5_ and Cu_3_Sn intermetallic compounds under micro-cantilever bending. Intermetallics.

[9] Zhang H., Dai J., Cao Y., Zhang Y., Bao M., Yin Y. (2024). A first-principles study of the mechanical and physical properties of Ni_3_Sn_x_ intermetallic compounds for high-temperature power device packaging. Intermetallics.

[10] Yang M., Chen J., Yang J., Zhang P., Yu Z., Zeng Z., Lu H. (2020). Interfacial transfer and phase evolution between Cu and Sn solder doped with minor Cu, Ag and Ni: Experimental and theoretical investigations. Appl. Phys. A.

[11] Emadi F., Vuorinen V., Mertin S., Widell K., Paulasto-Kröckel M. (2022). Microstructural and mechanical characterization of Cu/Sn SLID bonding utilizing Co as contact metallization layer. J. Alloys Compd..

[12] Han Y., Chen J., Lin M., Zhang K., Lu H. (2023). Synergistic effects of alloy elements on the structural stability, mechanical properties and electronic structure of Ni_3_Sn_4_: Using first principles. Vacuum.

[13] Kresse G., Furthmüller J. (1996). Efficiency of ab-initio total energy calculations for metals and semiconductors using a plane-wave basis set. Comput. Mater. Sci..

[14] Blöchl P.E. (1994). Projector augmented-wave method. Phys. Rev. B.

[15] Perdew J.P., Burke K., Ernzerhof M. (1996). Generalized Gradient Approximation Made Simple. Phys. Rev. Lett..

[16] He X., Zhu Y., Epstein A., Mo Y. (2018). Statistical variances of diffusional properties from ab initio molecular dynamics simulations. npj Comput. Mater..

[17] Gonze X., Lee C. (1997). Dynamical matrices, Born effective charges, dielectric permittivity tensors, and interatomic force constants from density-functional perturbation theory. Phys. Rev. B Condens. Matter Mater. Phys..

[18] Togo A., Tanaka I. (2015). First principles phonon calculations in materials science. Scr. Mater..

[19] Huang H.-L., Li G., Xiao X., Lu S.-Q., Peng P. (2020). Micromechanism in fracture toughness of NbCr_2_ laves phase improved by nickel alloying: First-principles calculation. J. Alloys Compd..

[20] Pan Y., Pu D., Liu G., Wang P. (2020). Influence of alloying elements on the structural stability, elastic, hardness and thermodynamic properties of Mo_5_SiB_2_ from first-principles calculations. Ceram. Int..

[21] Feng H.-L., Huang J.-H., Yang J., Zhou S.-K., Zhang R., Wang Y., Chen S.-H. (2017). Investigation of microstructural evolution and electrical properties for Ni-Sn transient liquid-phase sintering bonding. Electron. Mater. Lett..

[22] Li L., Weidner D.J., Brodholt J., Alfè D., Price G.D., Caracas R., Wentzcovitch R. (2006). Phase stability of CaSiO_3_ perovskite at high pressure and temperature: Insights from ab initio molecular dynamics. Phys. Earth Planet. Inter..

[23] Skripnyak N.V., Ponomareva A.V., Belov M.P., Syutkin E.A., Khvan A.V., Dinsdale A.T., Abrikosov I.A. (2020). Mixing enthalpies of alloys with dynamical instability: bcc Ti-V system. Acta Mater..

[24] Yang J.-W., An L., Zheng J.-J. (2021). Structure, mechanical and phonon stability of the Th-Sn system from ab initio. J. Nucl. Mater..

[25] Dai X., Savrasov S.Y., Kotliar G., Migliori A., Ledbetter H., Abrahams E. (2003). Calculated Phonon Spectra of Plutonium at High Temperatures. Science.

[26] Voigt W. (1889). Ueber die Beziehung zwischen den beiden Elasticitätsconstanten isotroper Körper. Ann. Der Phys..

[27] Reuss A. (1929). Berechnung der Fließgrenze von Mischkristallen auf Grund der Plastizitätsbedingung für Einkristalle. ZAMM J. Appl. Math. Mech./Z. Angew. Math. Mech..

[28] Hill R. (1952). The Elastic Behaviour of a Crystalline Aggregate. Proc. Phys. Soc. Sect. A.

[29] Mouhat F., Coudert F.-X. (2014). Necessary and sufficient elastic stability conditions in various crystal systems. Phys. Rev. B.

[30] Pugh S.F. (1954). XCII. Relations between the elastic moduli and the plastic properties of polycrystalline pure metals. Lond. Edinb. Dublin Philos. Mag. J. Sci..

[31] Li C.M., Zeng S.M., Chen Z.Q., Cheng N.P., Chen T.X. (2014). First-principles calculations of elastic and thermodynamic properties of the four main intermetallic phases in Al–Zn–Mg–Cu alloys. Comput. Mater. Sci..

[32] Ganeshan S., Shang S.L., Wang Y., Liu Z.K. (2009). Effect of alloying elements on the elastic properties of Mg from first-principles calculations. Acta Mater..

[33] Zhang H., Shang S.L., Wang Y., Saengdeejing A., Chen L.Q., Liu Z.K. (2010). First-principles calculations of the elastic, phonon and thermodynamic properties of Al_12_Mg_17_. Acta Mater..

[34] Nielsen O.H., Martin R.M. (1985). Quantum-mechanical theory of stress and force. Phys. Rev. B Condens. Matter.

[35] Niu H., Niu S., Oganov A.R. (2019). Simple and accurate model of fracture toughness of solids. J. Appl. Phys..

[36] Li L.H., Wang W.L., Wei B. (2015). First-principle and molecular dynamics calculations for physical properties of Ni–Sn alloy system. Comput. Mater. Sci..

[37] Marmier A., Lethbridge Z.A.D., Walton R.I., Smith C.W., Parker S.C., Evans K.E. (2010). ElAM: A computer program for the analysis and representation of anisotropic elastic properties. Comput. Phys. Commun..

[38] Yang C., Hu C., Xiang C., Nie H., Gu X., Xie L., He J., Zhang W., Yu Z., Luo J. (2021). Interfacial superstructures and chemical bonding transitions at metal-ceramic interfaces. Sci. Adv..

[39] Xing H., Dong A., Huang J., Zhang J., Sun B. (2018). Revisiting intrinsic brittleness and deformation behavior of B2 NiAl intermetallic compound: A first-principles study. J. Mater. Sci. Technol..

[40] Long Q., Nie X., Shang S.-L., Wang J., Du Y., Jin Z., Liu Z.-K. (2016). C15 NbCr_2_ Laves phase with mechanical properties beyond Pugh’s criterion. Comput. Mater. Sci..

[41] Zhang P., Chen M., Zhu Q., Zhang L., Fan G., Qin H., Tian Q. (2023). Micro Defects Evolution of Nickel-Based Single Crystal Superalloys during Shear Deformation: A Molecular Dynamics Study. Acta Metall. Sin. (Engl. Lett.).

[42] Kakehi K., Latief F.H., Sato T. (2014). Influence of primary and secondary orientations on creep rupture behavior of aluminized single crystal Ni-based superalloy. Mater. Sci. Eng. A.

